# Epigenetic programming of obesity in early life through modulation of the kynurenine pathway

**DOI:** 10.1038/s41366-024-01647-8

**Published:** 2024-10-18

**Authors:** Mojgan Gharipour, Jeffrey M. Craig, Garth Stephenson

**Affiliations:** 1 School of Medicine, Faculty of Health at Deakin University, Melbourne, VIC Australia; 2 IMPACT - the Institute for Mental and Physical Health and Clinical Translation, School of Medicine, Deakin University, Geelong, VIC Australia; 3 Murdoch Children’s Research Institute, Department of Pediatrics, The University of Melbourne, Royal Children’s Hospital, Melbourne, VIC Australia

**Keywords:** Cardiovascular diseases, Type 1 diabetes

## Abstract

Childhood obesity is a global health concern that has its origins before birth. Although genetics plays a crucial role, increasing evidence suggests that epigenetic modifications during fetal life could also influence its incidence. In this model, during the fetal period, interactions between genetic makeup, intrauterine factors, and environmental conditions, increase the risk of childhood obesity. This is in accordance with the Developmental Origins of Health and Disease (DOHaD) hypothesis, in which specific intrauterine environments can have long-lasting effects on the immune system’s essential functions during crucial stages of fetal growth, resulting in permanent changes to the immune function of the offspring. Consequently, dysfunction can consequently make the offspring more prone to inflammatory and immune-related disorders later in life. In this review, we examine how maternal inflammation could influence the risk of childhood obesity. We propose that during pregnancy, modification of the expression of critical genes in metabolic and signaling pathways, such as the kynurenine (Kyn) pathway, occurs due to increased levels of maternal inflammation. We also propose that such expression differences are mediated by epigenetic changes. Furthermore, we also hypothesize that the Kyn pathway produces metabolites that have immunoregulatory effects and may play a crucial role in regulating inflammation during pregnancy. As a result, interventions aimed at improving maternal inflammation may be able to help alleviate the risk of childhood obesity.

## Introduction

Childhood obesity is widely acknowledged as a significant global health concern due to its association with various adverse health outcomes [1]. Epidemiological studies have revealed a significant relationship between childhood obesity and factors such as sedentary lifestyles and unhealthy nutrition [2]. However, it is crucial to uncover the missing pieces of this puzzle to gain a better understanding of the relationship between life-threatening pregnancy complications and childhood obesity [3].

It’s widely known that gestational diabetes and gestational hypertensive disorders are associated with offspring obesity [4–6]. Additionally, fetuses of mothers with obesity are at risk of childhood obesity and heart disease later in life [6, 7]. Furthermore, studies of the Developmental Origins of Health and Disease (DOHaD) have shed light on epidemiological findings at the molecular level [8]. DOHaD research has shown a link between the periconceptual, fetal, and early infant life stages and the later onset of metabolic disorders through epigenetic modifications occurring in fetal/neonatal DNA due to influences from the maternal environment [9–11]. These modifications prompt the body to undergo epigenetic and physiological alterations, known as predictive adaptations, in anticipation of future conditions. This hypothesis eloquently describes how adverse intrauterine environments during the fetal period can impact gene and fetal programming without altering the DNA composition [12].

Epigenetic mechanisms such as DNA methylation, covalent histone modifications, and non-coding RNA collaboratively regulate the epigenetic state and gene activity [13]. For example, de novo methylation of CpG islands occurs in specific genes responsible for early embryonic development, exhibiting varying levels of methylation in tissue-specific patterns [14].

## Long-lasting effects of maternal risk factors on offspring health

Pregnancy is a complex physiological process involving changes in the maternal immune state throughout gestation. The maternal immune state is a tightly orchestrated process designed to facilitate pregnancy, while protecting the mother and fetus from infection and from maternal immune responses against the allogeneic fetus. T helper (Th) cells play a crucial role in moderating immune responses in different phases of human pregnancy. Dysregulation in Th responses show association with multiple obstetrical complications. The first and third trimesters have been identified as Th1 proinflammatory states, whereas the second trimester is characterized as a Th2 anti-inflammatory state [15, 16]. During the peri-implantation period of the first trimester, the Th_1_ immune response is required to facilitate tissue remodeling and wound healing following trophoblast invasion of the endometrium in addition to increased angiogenesis to support trophoblasts. In the second trimester, the immune system switches to a Th2_,_ anti-inflammatory state [17]. Here, paternal antigens in the trophoblasts activate Th2 cells at the maternal-fetal junction leading to the infiltration of Th2 cells into the decidua basalis of the placenta and the release of anti-inflammatory cytokines, which provide protection for the placenta and developing fetus against opportunistic infection. Following completion of fetal development in the third trimester, the placenta induces a Th1 immune state in preparation for birth, which aids in the contraction of the uterus, expulsion of the baby and rejection of the placenta itself [18]. Therefore, pregnancy is considered a trigger for a systemic inflammatory response. This is supported by evidence demonstrating increased plasma concentrations of acute-phase inflammation biomarkers, such as C-reactive protein (CRP), and a total white blood cell (WBC) count in the upper reference limit during pregnancy, elevated by up to 36% (5.7–15.0 × 10^9^/L). This is largely due to the increased leukocyte levels including neutrophils and monocytes which can increase by 55 and 38%, respectively during various stages of inflammation [19]. Although inflammation is a component of innate immunity, which aims to promote tissue repair and restore homeostasis, excessive inflammation can compromise cellular and tissue function [20]. For example, it has been suggested that excessive inflammation during pregnancy may result in maternal immune activation (MIA), leading programming of the fetal immune pathways and epigenetic changes resulting in increased expression of neurodevelopmental disorders in childhood [21]. In addition, a bidirectional relationship between epigenetic modifications and an increased inflammatory response during pregnancy appears to exist [22]. For instance, changes in DNA methylation patterns have been linked to the development of inflammation-related conditions such as preeclampsia and gestational diabetes [23–25]. Conversely, inflammatory stimuli can induce epigenetic modifications in immune cells, leading to changes in gene expression and potentially impacting pregnancy outcomes [26, 27].

Although the number of studies investigating the relationship between maternal inflammation and cardiometabolic outcomes in offspring through epigenetic modification is limited, a study by Bidne et al. [24] provides valuable insight into this area. While this particular study does not directly examine cardiometabolic outcomes, it demonstrated that pregnant mice fed a Western diet (WD, Envigo TD.88137) versus those on a normal rodent chow diet (ND, Teklad Global 18% protein rodent diet) exhibited an increase in certain lysophosphatidylcholines containing specific fatty acids and increased lipid storage in mid-gestational placentas. These findings suggest potential epigenetic mechanisms that could influence cardiometabolic outcomes, although further research is needed to establish this connection. Interestingly, despite these changes, no differences were observed in nutrient transport or fatty acid metabolism, suggesting that maternal obesity can impact placental lipid metabolism during early pregnancy, potentially leading to adverse pregnancy outcomes [28]. Another study in rats demonstrated that during fetal development, the expression of *Igf2* is regulated by maternal nutrient intake, potentially leading to disease phenotypes such as obesity. Gestational choline deficiency triggers epigenomic responses and may induce long-term developmental changes by modulating the fetal DNA methylation machinery. This complex process includes hypomethylation of regulatory CpGs within the *Dnmt1* gene, leading to its overexpression and subsequent elevated global and gene-specific (e.g., *Igf2*) DNA methylation [25]. A human study, by Rasool et al., found that in the first trimester, obesity is associated with the downregulation of lipid metabolism genes such as *ACOX1, CPT2, AMPKα, LPL, LIPG, MFSD2A, ACACA*, and *PLIN2*, which were significantly reduced in the placentas of women with obesity compared to those of lean women [29]. Furthermore, maternal obesity is associated with epigenetic changes in the leptin and adiponectin systems, shedding light on the molecular mechanisms involved in the placenta’s adaptation to a harmful maternal environment [30]. Additionally, elevated circulating lipids in pregnant mice with obesity have been observed to cross the placenta through specific fatty acid transporters [30]. Maternal obesity was also associated with epigenetic changes in genes of the leptin and adiponectin systems [31]. This suggests that the molecular mechanisms involved in the placenta’s adaptation to a harmful maternal environment may interfere with the hypothalamic expression of leptin receptors and pro-opiomelanocortin (*POMC*), activating inflammatory signaling, levels of increasing proinflammatory cytokines and oxidative stress within the placenta [32].

Another example of epigenetic modification in rat offspring due to changes in maternal diet is related to a protein-restricted diet during pregnancy, which can epigenetically change key adipogenesis genes. For example, the activation of peroxisomal proliferator-activated receptor-α (*PPAR-α*), an energy homeostasis gene, in the heart of the offspring contributes to maternal nutrition deficiency-led cardiomyopathy [33]. In addition, maternal low-protein diets in mice can regulate microRNA expression, which are associated with chronic inflammation status and metabolic health in offspring [34]. During fetal development, the expression of *IGF2* is regulated by maternal nutrient intake, potentially resulting in disease phenotypes, including obesity [23]. For example, in rats, gestational choline deficiency in diet initiates epigenomic responses and may supply the long-term developmental changes by modulating fetal DNA methylation machinery in a complex fashion that includes hypomethylation of the regulatory CpGs within the *Dnmt1* gene, leading to its overexpression and the subsequent elevated global and gene-specific (e.g. *Igf2*) DNA methylation [35].

In susceptible pregnant mothers, oxidative stress and circulating factors derived from the stressed placenta cause a systemic maternal inflammatory response, immune activation, endothelial cell dysfunction and vasospasm [36]. Inflammation during pregnancy is tightly related to support fetal growth and development by preventing impairment to the mother and fetus [37]. However, increasing levels of inflammation could be related to an increased risk of pregnancy complications, such as preterm birth, pre-eclampsia, and fetal growth restriction [38] Therefore, it is likely that increasing levels of pro-inflammatory cytokines, such as IL-1β, IL-6, and TNF-α, in the first to third trimester in the placenta could be related to the modification in the activity of the placental Kyn pathway [39]. These inflammatory cytokines, amongst others, have previously been identified in association with maternal immune activation (MIA) [40].

In line with our hypotheses, Ten et al. demonstrated that higher plasma concentrations of Kyn pathway metabolites are associated with obesity and increased risk for metabolic syndrome and fatty liver in prepubertal Asian children [41]. In addition, Dadvar et al. postulated that the activation of the Kyn metabolic pathway may also play a role in the development of obesity and metabolic dysfunction through the regulation of energy metabolism [42].

**Fig. 1:**
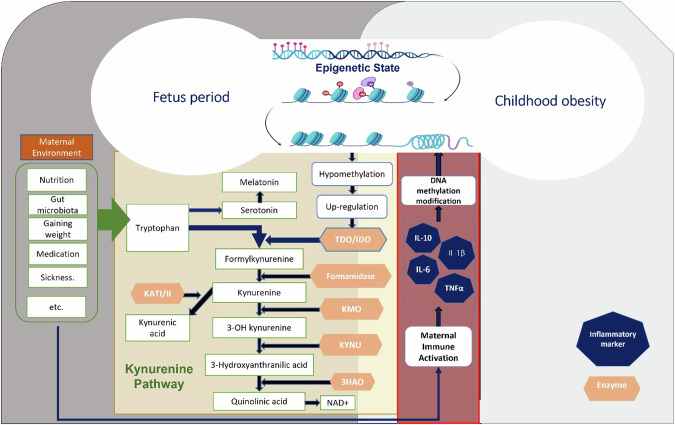
A hypothetical model of epigenetic programming of childhood obesity in early life through modulation of the kynurenine pathway. Environmental factors during pregnancy, such as nutritional habits, medication with an antidepressant, or composition of gut microbiota, modify the maternal immune system, changes the level of pro-inflammatory factors and induce modification in DNA methylation of *IDO* that led to changes in the level of IDO, and changes in levels of kynurenine metabolites. IDO Indoleamine 2, 3-dioxygenase, TDO Tryptophan 2,3-dioxygenase, KYNU Kynureninase, 3HAO 3-hydroxyanthranilate oxidase, KAT/II Kynurenine Aminotransferase Isozyme Inhibitor, KMO Kynurenine monooxygenase.

The KYN metabolic pathway is responsible for the metabolism of tryptophan, an essential amino acid required to produce serotonin, nicotinamide adenine dinucleotide (NAD), and other Kyn metabolites, such as neuroprotective (e.g., kynurenic acid) or neurotoxic (e.g., quinolinic acid), which play important roles such as vascular biology and neurological function [43], in addition to regulation of the immune system, and a wide range of pro-inflammatory biomarkers [44]. Tryptophan is an important essential amino acid critical to the function of several metabolites, including quinolinic and kynurenic acids. It cannot be produced by the body and therefore is obtained through the diet [45]. Within the placenta, a small proportion of the body’s tryptophan is converted to serotonin, which is essential for normal fetal brain development, and melatonin, which promotes survival and syncytialization of placental cytotrophoblasts while reducing free radicals and angiogenesis [46]. Most tryptophan enters the Kynurenine (Kyn) pathway and is converted to Kynurenine through a series of enzymatic steps. Tryptophan can also be converted to tryptamine through decarboxylation. Tryptamine can then undergo further enzymatic modification through transamination, resulting in the formation of indole pyruvic acid, which eventually leads to the production of kynurenine [47]. Kynurenine is further modified to produce biologically active metabolites, such as kynurenic acid and pro-inflammatory metabolites (Fig. 1). Kynurenine, when activated by the aryl hydrocarbon receptor (AHR), stimulates the expression of indol amine 2,3-dioxygenase 1 (IDO1)(41). It has been reported that a kynurenine ligand which binds to AHR is associated with the expression of anti-inflammatory genes, which may be disrupted by decreased methylation of *AHRR* (Aryl Hydrocarbon Receptor Repressor) [48]. In addition, Kyn also modulates reactive oxygen species (ROS) levels by signaling through the AHR [16].

## Physiological importance of the Kyn pathway in pregnancy

The Kyn pathway has various physiological roles that may be targeted to address several areas of aberrant pathophysiology observed in metabolic disease, oxidative stress, and immune activation. The pathway involves vitamin B6- and B2-dependent enzymes catalyzing the formation of kynurenines, including anthranilic acid, kynurenic acid, 3-hydroxykynurenine, 3-hydroxy anthranilic acid, and xanthurenic acid [49]. Furthermore, any external factors that could activate an inflammatory response in pregnancy could be related to the imbalance in Kyn pathway in pregnancy [50].

Inflammation can also activate the Kyn pathway to produce both neuroprotective and neurotoxic metabolites, depending on the balance of enzymes involved in the pathway [36]. In pregnancy, the Kyn pathway has been implicated in developing pre-eclampsia, a potentially severe complication characterized by high blood pressure and damage to multiple organ systems [51]. Some studies suggest that targeting the Kyn pathway may be a promising therapeutic strategy in management of pregnancy complications, as elevated levels of Kyn metabolites have been found in the blood and urine of subjects with preeclampsia. For instance, *IDO1* expression and activity in the maternal decidua and in the human placenta increases in a gradient towards the maternofetal interface, where *IDO1* activity is integral to the immunoregulation of normal pregnancy [52].

The maternal gut microbiota produces oxidative compounds, such as hydrogen peroxide, which suppress *IDO1* expression and can lead to maternal pro-inflammatory conditions [53]. This can shift the path of tryptophan catabolism from conversion to serotonin and melatonin to Kyn production, increasing the risk of fetal growth restriction and neurodevelopmental disorders [54]. We hypothesize that epigenetic modification in the activity of the Kyn pathway changes specific features of fetal metabolic pathways that could be related to obesity in later life. Therefore, understanding the role of the Kyn pathway in maternal inflammation and childhood obesity may provide an opportunity to develop novel therapeutic interventions for this condition. More research is needed to fully understand the complexity of the Kyn pathway and its role in obesity and related conditions.

## Conclusion

The development of obesity likely begins before birth, influenced by adverse intrauterine environments. These factors can result in epigenetic modifications that alter gene programming, leading to long-term changes in inflammatory pathways. Based on current evidence, we hypothesize that epigenetic modifications in regulatory genes of the Kyn pathway may play a crucial role in regulating inflammation during fetal life. These changes in gene expression could be associated with an increased risk of obesity later in life. Therefore, identifying novel epigenetic biomarkers as preventive therapeutic targets and implementing interventions aimed at improving maternal inflammatory factors may help reduce the risk of childhood obesity.
